# 7-T MRI of explanted liver and *ex-vivo* pancreatic specimens: prospective study protocol of radiological-pathological correlation feasibility (the EXLIPSE project)

**DOI:** 10.1186/s41747-020-00185-y

**Published:** 2020-10-15

**Authors:** Rosa Cervelli, Matteo Cencini, Guido Buonincontri, Francesco Campana, Andrea Cacciato Insilla, Giacomo Aringhieri, Paolo De Simone, Ugo Boggi, Daniela Campani, Michela Tosetti, Laura Crocetti

**Affiliations:** 1 Division of Diagnostic and Interventional Radiology, University of Pisa, Via Paradisa, 2 – Cisanello Hospital, 56100 Pisa, Italy; 2 IMAGO7 Foundation - IRCCS Stella Maris, Pisa, Italy; 3 Division of Pathology, University of Pisa, Pisa, Italy; 4 Division of Hepatic Surgery and Liver Transplant, University of Pisa, Pisa, Italy; 5 Division of General and Transplant and Surgery, University of Pisa, Pisa, Italy

**Keywords:** Liver cirrhosis, Liver steatosis, Liver transplantation, Magnetic resonance imaging, Pancreatic neoplasms

## Abstract

The study focuses on radiological-pathological correlation between imaging of *ex vivo* samples obtained by a 7-T scanner and histological examination. The specimens will be derived from native explanted cirrhotic livers, liver grafts excluded from donation because of severe steatosis, and primary pancreatic tumours. Magnetic resonance imaging (MRI) examinations will be performed within 24 h from liver or pancreatic lesion surgical removal. The MRI protocol will include morphological sequences, quantitative T1, T2, and fat-, water-fraction maps with Cartesian *k*-space acquisition, and multiparametric methods based on a transient-state “MRI fingerprinting”. Finally, the specimen will be fixed by formalin. Qualitative imaging analysis will be performed by two independent blinded radiologists to assess image consistency score. Quantitative analysis will be performed by drawing regions of interest on different tissue zones to measure T1 and T2 relaxation times as well as fat- and water-fraction. The same tissue areas will be analysed by the pathologists. This study will provide the possibility to improve our knowledge about qualitative and quantitative abdominal imaging assessment at 7 T, by correlating imaging characteristics and the corresponding histological composition of *ex vivo* specimens, in order to identify imaging biomarkers. Trial registration: ClinicalTrials.gov: 13646. Registered 9 July 2019—retrospectively registered

## Key points

- Explanted livers and pancreatic specimens will be investigated by 7-T magnetic resonance imaging (MRI).
- Morphological findings and quantitative data will be compared with specimen histology.
- Quantitative MRI will be explored for liver and pancreatic lesion characterisation.
- MRI fingerprinting will be used to estimate quantitative maps in a shorter time.

## Background

Magnetic resonance imaging (MRI) provides information on tissue structure and function by means of measurements of intrinsic differences in relaxation times of hydrogen (as well as other) nuclei following radiofrequency excitation. By increasing the magnetic field of the equipment, an intrinsic increase of the signal-to-noise ratio can be achieved [1, 2]. As a consequence, ultra-high magnetic field strengths such as that used in 7-T units may provide clinical advantages by improving quantitative map estimation, increasing the spatial resolution, or reducing the scan time.

This study focuses on the comparison between 7-T MRI of *ex vivo* samples and their histological examination, to assess the correlation between tissue imaging characteristics and pathological features. The examined samples will be native cirrhotic livers explanted during orthotopic liver transplantation (group A), liver grafts excluded from donation because of severe steatosis (group B), and surgical specimens of primary pancreatic tumours, which underwent upfront pancreaticoduodenectomy or total pancreatectomy (group C). To obtain the information on tissue structure and function, this study will include conventional methods and novel multiparametric quantitative evaluation. The novel multiparametric methods are based on a transient-state magnetic resonance fingerprinting (MRF) [3] sequence with spiral trajectory *k*-space acquisitions in two or three dimensions. While conventional imaging for quantitative mapping usually needs multiple acquisitions to obtain the measurements of the different parameters, MRF can provide multiple quantitative information with a single acquisition. This technique compares the variable signal acquired per each pixel during the acquisition with a predefined dictionary of signal evolution, allowing the recognition and the measurement of the different parameters based on the match of their signal evolution. Using MRF to evaluate the upper abdomen, including the liver and pancreas, Chen et al. [4] succeeded in the simultaneous quantification of T1 and T2 relaxation time values in the abdomen with a 19-s breath-hold acquisition.

The radiological-pathological correlation may improve the knowledge of the changes that occur in cirrhotic liver and their focal lesions (inflammatory, proliferative non-neoplastic, degenerative, and neoplastic lesions), in non-alcoholic steatotic liver disease, which represents one of the main cause of unsuitability for liver transplant liver graft, and pancreatic ductal adenocarcinoma (PDAC, group C). More in detail, the T1 and T2 relaxation time values and the fat fraction will be assessed on targeted tissues, consisting of focal pancreatic/hepatic lesions or liver parenchyma (for the excluded liver grafts) and of parenchymal tissues different from the targeted one such as non-neoplastic surrounding parenchyma (non-targeted tissues).

We assume that statistically significant differences in relaxation time values between the lesions and non-targeted tissue will be observed.

In fact, as already explored in different clinical fields, T1 and T2 relaxation time estimation provided promising results in oncologic and non-oncologic tissue characterisation and evaluation of neoplastic tissue changes after treatment [5]. Among these applications, quantitative MRI has been shown to be useful in the evaluation of tissue compositional changes occurring in liver fibrosis and hemosiderosis [6–8], as well as in the assessment of the liver fat content in the steatosis and steatohepatitis scenarios [8, 9]. Similarly, pancreatic parenchyma have been studied through quantitative MRI analysis in both acute and chronic pathological processes with promising identification of noninvasive imaging biomarkers, including T1 and T2 relaxation time values [10]. Further studies are needed to explore the role of quantitative MRI in understanding the underlying pathological tissue changes and processes.

The rationale behind the group A of native livers explanted from patients undergoing orthotopic liver transplantation derives from the possibility of investigating focal lesions focusing on hepatocellular carcinoma [11, 12] and the effect of locoregional therapy (microwave ablation, transarterial chemoembolisation, and ^90^Y transarterial radioembolisation), when applied as a “bridge treatment” [13]. All this information will be collected into a data bank to improve the knowledge of many conditions that affect liver candidates for transplant.

Group B will include grafts that are excluded from liver transplantation due to severe macrovesicular steatosis [14, 15]. Due to the limited availability of organs for liver transplant and long waiting lists for transplantation, many attempts were made to increase the number of donors, including the use of the “steatotic extended criteria donor” [16]. The risk of graft failure and dysfunction after transplantation of those livers is related to an increase risk in ischemia-reperfusion injury [17]. Thus, the group B of livers represent precious material to investigate the distribution of steatosis and the liver damage induced by it, in order to identify and select “good-steatotic livers” by 7-T MRI.

Finally, regarding PDAC, despite significant improvements in surgical and nonsurgical treatment modalities, including new possible therapeutic targets [18, 19], 5-year survival rate of 5% of this tumour remains almost unchanged. Therefore, reproducible preclinical models are required to study the growth pattern and local peritumoural environment of pancreatic cancer, to develop new and effective treatment modalities for *in vivo* applications. These considerations guide the aim of the study for group C: the study of *ex vivo* pancreatic surgical specimens by 7-T MRI should provide a reference framework to define proper acquisition protocols and imaging biomarkers for future clinical applications.

## Study aims

The main aim is to evaluate the correlation between the characteristics detected by the 7-T equipment and the histological composition of *ex vivo* specimens.

The primary study aims for groups A and B are to identify useful imaging biomarkers based on the evaluation of:

- Radiological-pathological correlation of focal lesions affecting the cirrhotic liver and of fibrotic/regenerative characteristics of the liver tissue, free from focal lesions; comparisons will include morphological matching as well as correlations between T1 and T2 relaxation time values and differential diagnosis between lesions and pseudolesions or histological classification of the fibrosis degree;
- Radiological-histological correlation of hepatic lesions treated by locoregional “bridge” treatments, in particular by evaluating the T1 or T2 relaxation time value difference between lesion histologically classified as complete responders and as partial responder after treatment;
- The degree of steatosis and to correlate this radiological grading with the well-established histological classification of the steatosis distribution [20];

The study aim for group C is the following:

- Radiological-pathological correlation of pancreatic lesions; comparisons will include morphological matching between macroscopic histological evaluation and MRI as well as the correlations between T1 and T2 relaxation time values with the lesion extracellular microenvironment and lesion aggressiveness, respectively.

Secondary study aim is to standardise and validate new 7-T MRI protocols, including quantitative sequences, to be possibly implemented also on 3-T clinical scanners.

## Methods

This research protocol is a prospective experimental correlational no-profit study on human *ex vivo* specimens from liver and pancreatic tissues.

### Ethical issues and enrolment

Before starting the study, every participant of A and C groups will be accurately informed about the management of the resected specimen (whole native liver explanted during orthotopic liver transplantation or pancreatic specimens resected due to the presence of a PDAC lesion) and will be given the informative letter for them and for their general practitioner, the forms of the informed consent, and the one for the treatment of their data for research purposes. All participants will be registered after signing the informed consent. The patients’ eligibility criteria, based on the study group of enrolment, are summarised in Table 1. The informed consent to participate to the “EXLIPSE” research study does not apply to group B since the authorisation to organ donation in our country implies also the consent to investigate the explanted organs for scientific purposes.

**Table 1:** Inclusion and exclusion criteria according to the sample group

	Inclusion criteria	Exclusion criteria
Group “A” of native liver	- Age ≥ 18 years - Informed consent - Full possession of one’s own faculties - Patients who are going to undergo liver transplantation, independently from previous therapy or from the primary disease they are affected by	- Patients affected by polycystic liver disease - Excluded from liver transplantation
Group “B” of excluded grafts	- Liver grafts with histologically proven macrovesicular steatosis, not fit for transplantation	- Liver grafts histologically proven macrovesicular steatosis fit for transplantation - Liver grafts not fit for transplantation due to causes other than steatosis
Group “C” of focal pancreatic lesions	- Age ≥ 18 years - Informed consent - Full possession of one’s own faculties - Affected by pancreatic lesion histologically proven to be a carcinoma or with imaging and laboratory findings highly suspicious for PDAC, and treated by up-front pancreaticoduodenectomy or total pancreatectomy - Assessed by contrast-enhanced CT examination within 21 days before surgery	- Excluded from surgery due to the severity of their condition.

The study will be performed in agreement with the dictates of the Helsinki Declaration and the rules of Good Clinical Practice (E6: Good Clinical Practice: Consolidated Guideline (CPMP/ICH/135/95)). The approval of regional Ethical Committee to conduct this “single-centre prospective study” was obtained (protocol number approval: 13646).

The enrolment stage, as well as the analysis duration, will last 3 years.

### 7-T MRI

It is well established by the literature that an unavoidable signal difference between *ex vivo* and *in vivo* biological tissues is the one caused by fixation [21, 22]. In fact, fixation reduces both relaxation times and diffusivity. Thus, the specimens will be placed in a sterile, nonpyrogenic solution for hypothermic storage (Servator C®, SALF, Bergamo, Italy). Each specimen will be maintained at a temperature ranging between 4 and 8 °C in the solution, until the MRI examination is started.

During the MRI study, the specimen will be placed in a dedicated single-use plastic box previously disinfected by a 90% alcohol solution and dried by a sterile towel (Supplemental material, Fig. S1). The MRI acquisition protocol will start when the specimen reaches the temperature of 20 °C (in balance with the constantly monitored room temperature); that temperature increase allows the hydrogen-proton, positively charged, to turn its spin axis in order to generate the resonance signal phenomena. During the MRI examination, liver or pancreatic specimen temperature will range from 20 to 25 °C; if an over-range increase takes place, the examination will be interrupted. The temperature increase will be verified and monitored by the real-time estimation of specific absorption rate provided by the scanner, which allows the indirect estimation of the temperature increase. In addition, a MRI-compatible fibreoptic temperature probe will be used at the end of each sequence as further confirmation that the temperature limits are respected. Finally, the pathologist will identify any sign of specimen degeneration/contamination. Specimens with pathological signs of degeneration/contamination will be excluded.

To guarantee the correct storage of the specimens, so that they maintain all the physiological, histological characteristics, all the MRI examinations will be performed within 24 h from the liver or pancreatic lesion surgical removal. At the end of the MRI study, the *ex vivo* specimens will be again stored in the solution at 4 °C. Finally, the native explanted liver, the liver graft, and/or the primary pancreatic lesions will be fixed by formalin and analysed by the pathologist.

The imaging protocol will include both morphological and quantitative imaging acquisitions performed on a Discovery MR 950 7-T MRI scanner (General Electric Healthcare, Milwaukee - WI, USA) equipped with an autonomously customised 8-channel transmit/receive knee coil.

#### Morphological acquisitions

They will include a three-dimensional CUBE T2-weighted fast turbo spin-echo sequence as anatomical reference, as well as T1- and T2-weighted water-only sequences using the “iterative decomposition of water and fat with echo asymmetry and least-squares estimation” (IDEAL) technique [23] and two- and three-dimensional cholangiopancreatography acquisitions. Imaging parameters are listed in Table 2.

**Table 2:** Imaging parameters for both 7-T morphological and quantitative acquisitions

Parameter	3D CUBE	IDEAL T2-w	IDEAL T1-w	2D MRCP	3D MRCP	MP2-RAGE	MSE	IDEAL fat-water	2D MRF	3D MRF
Flip angle (°)	90	90	90	90	90	4	90	1	NA	NA
Inversion time (ms)	NA	NA	NA	NA	NA	1000; 3200	NA	NA	18	18
Echo time (ms)*	31.7	52.8	9.9	454	545	2.4	9:9:36; 13:13:52	NA	NA	0.455
Echo train length	20	15	7	NA	130	NA	NA	NA	NA	NA
Repetition time (ms)	1,000	3,700	345	10,000	4,000	6.3	2,500	5	NA	7.4
Bandwidth (kHz)	125	83	83	125	125	62.5	62.5	62.5		
Number of excitations	2	4	3	1	1	1	0.5	4	1	1
Field of view (mm)**	192 × 192	180 × 180/160 × 160	180 × 180/160 × 160	180 × 180/160 × 160	180 × 180/160 × 160	192 × 192	225 × 225	225 × 225	225 × 225	225 × 225
Slice thickness (mm)	0.6	1.5	1.5	3	1	1	5	5	5	1.125
Slice spacing (mm)	NA	0.3	0.3	0	NA	0	0	0	0	NA
Reconstruction matrix	512 × 512	512 × 512	512 × 512	512 × 512	512 × 512	256 × 256	256 × 256	256 × 256	192 × 192	200 × 200
Parallel imaging acceleration	2	NA	2	2	2	NA	NA	NA	NA	NA

*2D* Two-dimensional, *3D* Three dimensional, *T1-w.* T1-weighted, *T2-w.* T2-weighted, *IDEAL* Iterative decomposition of water and fat with echo asymmetry and least-squares estimation, *MP2RAGE* Modified magnetisation prepared rapid gradient echo acquisition, *MRF* Magnetic resonance fingerprinting, *MRCP* Magnetic resonance cholangiopancreatography, *MSE* Multiple spin-echo

*For MSE, the echo time notation is “minimum:step:maximum”

**Field of view for T1-w./T2-w. IDEAL and 2D/3D MRCP were 180 mm × 180 mm for liver specimens (groups A and B) and 160 mm × 160 mm for pancreatic specimens (group C)

*Quantitative mapping* will include the following series of techniques and methods.

#### T1 mapping

T1 values will be obtained using a modified magnetisation-prepared rapid gradient echo acquisition, named MP2RAGE. Briefly, it consists of the combination of two low-flip angle images with different inversion times (TI). These images are combined according to the following equation

1 $$ \mathrm{MP}2\mathrm{RAGE}\ \mathrm{intensity}=\frac{\mathrm{T}{\mathrm{I}}_1\mathrm{T}{\mathrm{I}}_2}{\mathrm{T}{\mathrm{I}}_1^2+\mathrm{T}{\mathrm{I}}_2^2} $$

where TI_1, 2_ are the two images with different TIs (TI_1_ and TI_2_). This combined image, due to the low flip angle, results to be bias corrected. Moreover, exploiting the knowledge of the acquisition parameters, the T1 values can be uniquely determined from the intensity values as previously described [24].

#### T2 mapping

T2 values will be obtained by multi-echo spin-echo acquisition. Images for the individual echoes are used to obtain the T2 map using the following signal model:

2 $$ \ln \left(S\left(\mathrm{TE}\right)\right)=\ln \left({S}_0\right)-\frac{\mathrm{TE}}{T_2} $$

where *S*(TE) represents the voxel-wise signal magnitude for the echo time (TE).

#### Fat fraction mapping

Fat fraction values will be obtained from an IDEAL fast gradient-echo acquisition. To avoid T1-weighting and obtain a proton density-weighted fat fraction, a low flip angle is used. Output water and fat images are combined to obtain fat fraction map, as follows:

3 $$ \mathrm{FF}=\frac{F}{W+F} $$

where *F* and *W* represent the signal of fat and water, respectively.

#### Multiparametric techniques (MRF)

**Fig. 1:**
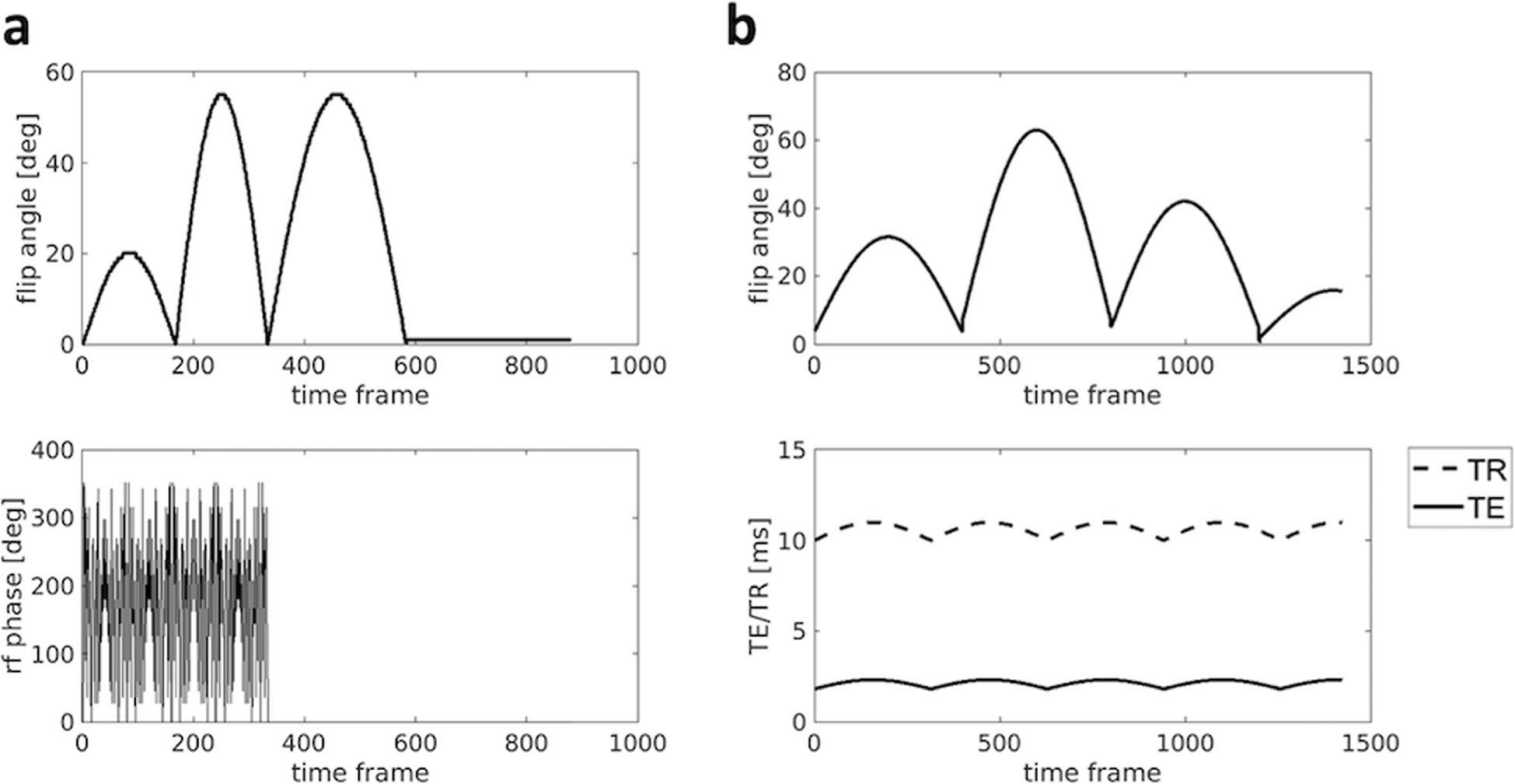
**a** Flip angle schedule for three-dimensional magnetic resonance fingerprinting (top row) and radiofrequency phase schedule (bottom row). **b** Flip angle schedule (top row) and echo time (TE)/repetition time (TR) schedule (bottom row) for two-dimensional magnetic resonance fingerprinting; a radiofrequency (rf) spoiling with quadratic phase increment of 117° is used during the whole acquisition

Multiparametric acquisitions in the transient state *(i.e.*, preventing the magnetisation from reaching an equilibrium condition by continuously changing the acquisition parameters between each readout) will be included in the protocol. Two different MRF acquisitions will be performed: a three-dimensional acquisition based on spiral projections, which will provide T1, T2, and B_1_+ transmit field maps, and a spiral two-dimensional acquisition, which will provide T1, FF, B_0_, and B_1_+ transmit field maps. In both cases, the parametric maps will be obtained by matching the acquired data to a precomputed dictionary of signal evolutions obtained by means of numerical simulations. For two-dimensional MRF, parameter inference will be performed in two steps [25], where B_0_ values are estimated in the first step and used as input parameter for the second step, where FF, water T1, and B_1_+ are obtained. Variation of flip angle and other technical parameters are shown in Fig. 1, while dictionary input parameters are listed in Table 3.

**Table 3:** Values for dictionary creation

	Water T1 [ms]	Fat T1 [ms]	T2 [ms]	B1+ [a.u.]	B0 [Hz]	Fat fraction
Dictionary 1 (2D MRF–step 1)	0:300:4000	500	NA	0.1:0.2:0.9; 0.9:0.2:2	-500:5:500	0:0.1:1
Dictionary 2 (2D MRF–step 2)	0:5:1550; 1600:100:4000	500	NA	0:0.1:2	0	0:0.05:1
Dictionary 3 (3D MRF)	20:10:3000; 3100:50:4500	NA	1:1:30; 35:5:50	0:0.1:2	NA	NA

### Outcome measures

Qualitative and quantitative analyses of all collected images will be performed at dedicated workstations (ADW 4.4 and 4.6, General Electric Healthcare, Milwaukee-WI, USA).

#### Qualitative image analysis

Two independent blinded readers (board-certified radiologists with 5- and 15-year experience in abdominal imaging) will assess an image consistency score on a 4-point scale depending on different factors (image signal intensity, imaging resolution, imaging noise, field homogeneity, radiologist’s diagnostic confidence in relation to imaging findings), as follows: 1 = poor, insufficient image quality (radiologist not confident at all to propose a diagnosis); 2 = sufficient (it is possible to propose a diagnostic hypothesis but the image quality does not allow a reliable diagnosis); 3 = good (good image quality, which allows to propose a reliable diagnosis); 4 = excellent (good/excellent image quality with excellent depiction of morphologic details, associated with image characteristics which allows the radiologist to be very confident about the diagnosis).

#### Quantitative analyses

It will require previously defined regions of interest (ROIs) and cannot be assessed in the whole organ/specimen—will be performed on different areas: (a) “targeted tissue” (group B) or “targeted lesion” (groups A and C); (b) tissue close to the targeted ones but above 5-mm distance from the target (non-target tissue). The non-target tissue refers to a parenchymal area not involved in focal alteration/lesion, but with tissue changes related to other systemic diseases such as fibrosis in cirrhotic livers. In groups A and C, the targeted lesion will be measured including the whole focal lesion, by drawing a hand-made ROI along lesion edges and (when allowed by lesion size) on the lesion central and peripheral zones by circular ROIs. In the latter cases, the area of each ROI will include more than 100 voxels. In order to reduce measurement error, the measurement will be repeated three times, and both single and mean values will be analysed.

#### Radiological-pathological correlation

The same tissue areas will be analysed by the pathologists, thanks to the presence of the anatomical landmarks identified by the radiologist during the MRI examination and referred to the pathologist. She/he will be able to recognise by using the same section plan defined for imaging and histological analysis. In fact, the section of the two-dimensional acquisition plane will be defined together with the pathologists in order to perform the subsequent histological analysis of the same section of the tissue. Unfortunately, the MRI slice thickness will not be the same of the histological slice; as a consequence, the difference in slice thickness will remain a limit of our study. The expected outcomes of radiological-pathological correlation are summarised in Table 4.

**Table 4:** Radiological-pathological comparisons and expected outcomes

Analysis	Imaging investigated	Pathological reference	Expected outcomes
**Qualitative analyses**			
1. Image consistency	CUBE IDEAL T1w IDEAL T2w IDEAL Fat-water 2D MRCP 3D MRCP MP2RAGE MSE 3D MRF 2D MRF	Histological confirmation of the presence and type of lesion/tissue alteration identified by imaging	Diagnostic reliability using morphological sequences
**Quantitative analyses**			
1. T1 relaxation time values of cirrhotic liver parenchyma 2. T1 relaxation time value of hepatic lesions 3. T1 relaxation time value of pancreatic lesion	MP2RAGE 3D MRF (T1-map) 2D MRF (T1-map)	1. Fibrotic/regenerative characteristics of the liver tissue 2. Focal lesions affecting the cirrhotic liver 3. Pancreatic lesion characteristics	1. Correlation between the fibrotic degree and the T1-value 2. Differential diagnosis of the focal hepatic lesions based on T1-value 3. Pancreatic lesion stroma related to T1-value
1. T2 relaxation time values of hepatic lesions 2. T2 relaxation time value of pancreatic lesion	MSE 3D MRF (T2-map)	1. Focal lesions affecting the cirrhotic liver 2. Pancreatic lesion characteristics	1. Differential diagnosis of the focal hepatic lesions based on T2-value 2. Pancreatic lesion aggressiveness related to T2-value
Fat-fraction values of steatotic liver parenchyma	IDEAL Fat-water 2D MRF (fat-fraction and water-fraction) 3D MRF (fat-fraction and water-fraction)	Well-established histological classification of the steatosis distribution	Good radiological-pathological correlation about the degree of steatosis

#### Pathological analysis

After the MRI study, two pathologists with 20- and 5-year experience in hepatobiliopancreatic pathologies will analyse all specimens. First, the pathologists will evaluate the whole specimen; then, they will focus on specific tissue slices corresponding to the ones on which the radiologists will provide their diagnosis and diagnostic confidence score.

For group A, gross examination of the liver will be performed reporting on the size and weight of the specimen as well as the appearance of its external surface. In particular, cirrhosis will be described according to the dimension of single nodules as micronodular (< 3 mm) or macronodular (> 3 cm). Subsequently, the liver will be dissected; colour and consistency of liver parenchyma will be described as well as any suspect nodule. Representative samples will be taken for microscopic examination. Main histological features will be reported, and liver neoplasms, when present, will be reported and described according to the 2019 World Health Organization classification of digestive system tumours [26].

For group B, gross examination and dissection will be performed according to the same protocol described for group A. Steatosis will be determined on light microscopy, in formalin-fixed, paraffin-embedded sections and will be described as macrovesicular, in case of hepatocytes distended by a single droplet/few droplets, which displace the nucleus, or microvesicular, in case of swollen hepatocytes containing very small vacuoles, without displacement of the nucleus. In addition, the percentage of hepatocytes with steatosis as well as their localisation (pericentral, lobular, periportal) will be reported.

For group C, before sampling the specimen, a multicoloured inking of all the pancreatic circumferential margins will be performed. The pancreatic head will be sliced along the axial plane, perpendicularly to the descending part of the duodenum, with a dissection plane similar to that of MRI. Dissection will include the entire head of the pancreas and any adherent structures such as duodenal wall, distal common bile duct, and vascular resection, when present. All main histological features of the tumour and collateral tissue will be reported during the microscopic examination. Margins will be defined negative when no tumour cells are found within 1 mm from inked margins [27].

### Sample size and statistical analysis

To the best of our knowledge, the EXLIPSE study could be the first study on quantitative 7-T MRI of *ex vivo* human liver and pancreatic specimens. As a consequence, the sample size calculation cannot be based on previous published experiences. Thus, this preliminary study will start by including at least ten *ex vivo* specimens per group, a sample size considered as appropriate in terms of feasibility assessment.

Before performing inferential tests, Kolmogorov-Smirnov’s test will be used to evaluate data distribution. In the case of normal distribution, the quantitative T1 and T2 values inside the ROIs obtained will be compared with the different degree assigned to the examined histological tissue characteristics (fibrotic grade, fat content, and pancreatic cancer marker of aggressiveness, such as involvement of circumferential resection margins) using a mixed effects ANOVA model. By this statistics, it will be possible to identify differences among the different groups. In the case of not normal distribution, they will be compared by using the Kruskal-Wallis test. To assess the correlation between the relaxation time values and histological measurements Pearson *r* or Spearman *ρ* will be applied, according to data distribution. When the ANOVA test includes several groups and the analysis results are significant, a post hoc analysis will be performed applying the Bonferroni correction

The *p* values will be calculated using the Chi-square distribution for nominal variables. Thus, the correlation between the morphological MRI data and the information provided by the macroscopic and microscopic histological examination of the specimens will be performed. Specimen characteristics will be summarised using descriptive statistics: mean and standard deviation, or median and range for continuous variables, in the case of normal or not normal distribution, respectively, absolute and percentage frequencies for categorical variables.

A *p* value lower than 0.05 will be considered statistically significant, with exception of cases when Bonferroni correction will be appropriate.

## Discussion

The use of specimens allows a high spatial resolution without motion artefacts to guarantee a precise comparison between the information obtained by image analysis and the specimen macroscopic and microscopic pathological examinations. The effort made to build this study protocol was driven by the belief that *ex vivo* specimens investigated by 7-T MRI allow a reliable comparison between radiological images and histology. Moreover, 7-T MRI has already been demonstrated as a useful tool able to provide information on surgical margins after surgery. In particular, 7-T MRI showed a good correlation with pathology in the evaluation of negative surgical margins after partial nephrectomy with and sensitivity and specificity for MR assessment of negative surgical margins of 100% and 75%, respectively. With a mandatory protocol optimisation to minimise the scan time, the authors proposed the 7-T MRI specimen evaluation as a promising intraoperative tool to improve the surgical outcomes about margins and parenchymal sparing [28]. As already demonstrated in other anatomical districts, the comparison between 7-T MRI and pathology might identify previously undefined quantitative imaging biomarkers and might recognise morphological changes able to give information for the diagnosis and management of many systemic (cirrhosis and steatosis) and focal hepatic diseases, in particular cancer (PDAC and hepatocellular carcinoma).

Due to the small study sample per group, the identified imaging biomarkers and morphological tissue changes will have to be validated by future dedicated studies, which will be conducted both on a larger *ex vivo* sample and on an *in vivo* population.

This research protocol had to overcome some difficulties in its design. First, specimen storage: the pathologist responsible for the analysis of liver and pancreas specimens was asked to evaluate how to store the specimens before histological analysis. The specimens should not be treated with formalin before 7-T MRI because a decrease in magnetic resonance tissue characteristics including T1 and T2 relaxation times have been demonstrated after fixation [22, 29–31].

Thus, the pathologists assessed that no tissue damage occurs if the time length from surgical resection to MRI exam and to delivery to the pathologist is less than 24 h. During these 24 h, the specimen should be maintained at a temperature of 4 °C, with the exception of the time spent in the MRI magnet when the specimen temperature will arise above 15 °C. This temperature will be controlled during the exam so that the heating related to radiofrequency exposure does not damage the tissue. The exam will be interrupted if the temperature exceeds 25 °C. Second, the logistics: the lab where the 7-T device is located is about 15 km from the surgical suites and from pathology department. For this reason, the transportation of the specimens required dedicated medical vehicles. Finally, the lack of previous similar experiences in the literature: the 7-T knee coil has been predominantly used and optimised for knee investigations; thus, no examples of technical parameters to study *ex vivo* specimens were available. As a consequence, before starting the EXLIPSE study, an acquisition protocol had to be set-up.

This study will provide the possibility to improve our knowledge about the quantitative abdominal imaging assessment. In fact, the EXLIPSE study could represent an important step to further develop MRF sequences on 3-T MRI. The advantage of using 7-T radiological-pathological correlation to optimise MRF consists in the opportunity of employing the tissue morphologic and quantitative characteristics discovered by the ultrahigh field and confirmed by histology as a reference standard for constructing the signal on the similar sequences for 3-T systems. We expect that the higher 7-T signal in the quantitative maps could provide a useful information, even if for relatively small samples. The higher resolution could provide a more suitable correlation with micron-size histology evaluation.

Studies at lower field strengths could benefit from larger samples to achieve further results. Once imaging biomarkers are identified, the next step will be to exploit this knowledge for application on 3-T device, to create a more widespread demonstration and application of their clinical utility, due to the remarkably larger availability of the 3-T scanner. Protocol and sequence optimisation process will be mandatory at 3 T due to the different contrast (increased T1 and decreased T2 at 7 T than at 3 T), potential artefacts observed on the images obtained at different static magnetic fields, and the different availability of dedicated coils. However, the presence of well-optimised dedicated coils for abdominal imaging at 3 T may lead to a more rapid optimisation of sequences and protocols.

## Supplementary information

**Additional file 1.**

## Data Availability

The datasets used and/or analysed during the current study are available from the corresponding author on reasonable request.

## Abbreviations

ANOVA: Analysis of variance
IDEAL: Iterative decomposition of water and fat with echo asymmetry and least-squares estimation
MRF: Magnetic resonance fingerprinting
MRI: Magnetic resonance imaging
PDAC: Pancreatic ductal adenocarcinoma
ROI: Region of interest
