# Supplementary figures and images for: 7-T MRI of explanted liver and ex-vivo pancreatic specimens: prospective study protocol of radiological-pathological correlation feasibility (the EXLIPSE project)
Source: Eur Radiol Exp. 2020 Oct 15;4:58. doi: 10.1186/s41747-020-00185-y (PMC7560686; doi:10.1186/s41747-020-00185-y)

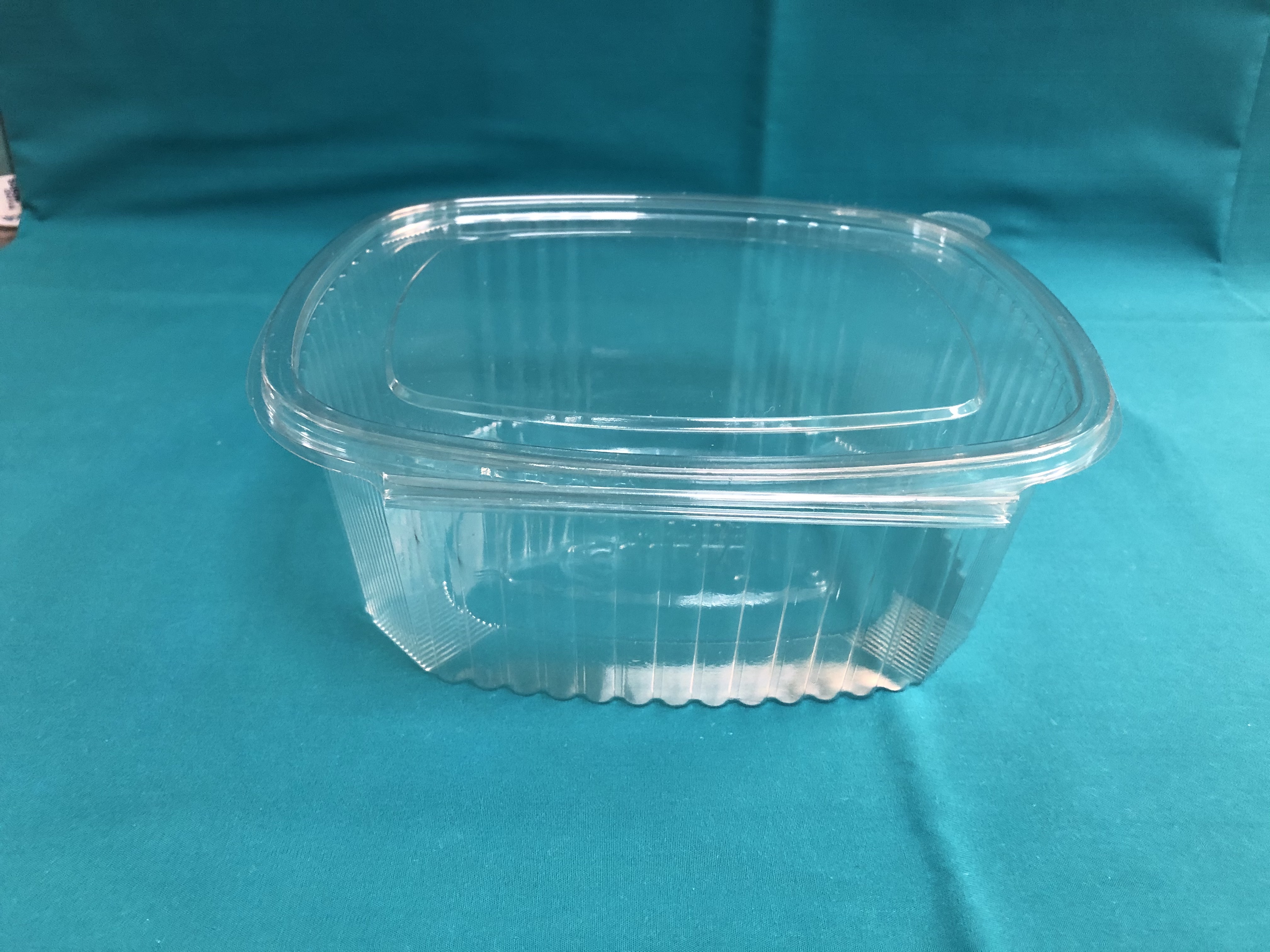

Supplement: Supplementary file 1 — Additional file 1. [file 41747_2020_185_MOESM1_ESM.jpg]
